# Acute Lymphoblastic Leukemia in a Young Adult Presenting as Hepatitis and Acute Kidney Injury

**DOI:** 10.1177/2324709616665866

**Published:** 2016-09-22

**Authors:** Marc Heincelman, Nithin Karakala, Don C. Rockey

**Affiliations:** 1 Medical University of South Carolina, Charleston, SC, USA

**Keywords:** differential diagnosis, liver, aminotransferase, drug induced liver injury, deep vein thrombosis

## Abstract

Acute lymphoblastic leukemia (ALL) in adults is a relatively rare malignancy. The typical presentation includes signs and symptoms associated with bone marrow failure, including fevers, infections, fatigue, and excessive bruising. In this article, we report an unusual systemic presentation of ALL in a previously healthy 18-year-old man. He initially presented with several-day history of nausea and vomiting, 10-pound weight loss, and right upper quadrant abdominal pain with evidence of acute hepatocellular liver injury (elevations in aspartate aminotransferase/alanine aminotransferase) and elevation in serum creatinine. Further history revealed that he just joined the Marine Corp; in preparation, he had been lifting weights and taking protein and creatine supplements. A complete serological evaluation for liver disease was negative and creatine phosphokinase was normal. His aspartate aminotransferase and alanine aminotransferase declined, and he was discharged with expected improvement. However, he returned one week later with continued symptoms and greater elevation of aminotransferases. Liver biopsy was nondiagnostic, revealing scattered portal and lobular inflammatory cells (primarily lymphocytes) felt to be consistent with drug-induced liver injury or viral hepatitis. Given his elevated creatinine, unresponsive to aggressive volume expansion, a kidney biopsy was performed, revealing normal histology. He subsequently developed an extensive left lower extremity deep venous thrombosis. Given his deep venous thrombosis, his peripheral blood was sent for flow cytometry, which revealed lymphoblasts. Bone marrow biopsy revealed 78% blasts with markers consistent with acute B-cell lymphoblastic leukemia. This report emphasizes that right upper quadrant abdominal pain with liver test abnormalities may be the initial presentation of a systemic illness such as ALL.

## Case Presentation

A previously healthy 18-year-old male presented with a 2-week history of decreased oral intake, nausea, vomiting, abdominal pain, and 10-pound weight loss. Emesis was nonbloody, nonbilious without hematemesis. Further history revealed that he recently moved from Michigan to South Carolina 3 weeks prior where he started boot camp after joining the Marine Corp. In preparation, he had been lifting weights and taking protein and creatine supplements. His symptoms correlated with the initiation of boot camp. There were no associated fevers, dysuria, or other symptoms.

He had been healthy his entire life with no past medical disorders or surgical procedures, and was not taking any medications. Social history revealed he was sexually active with women, occasionally without condom use. On examination, he was a thin male with mild muscle wasting. Vital signs were normal and he was comfortable appearing. His abdomen was soft with mild tenderness to deep palpation in the right upper quadrant without rebound or guarding. There were no stigmata of chronic liver disease and no scleral icterus.

Laboratory data revealed a white blood cell count 14 400/cm^3^ (34% neutrophils, 18% lymphocytes, 33% reactive lymphocytes, 2% eosinophils), hemoglobin 18.3 g/dL, hematocrit 52%, and platelet count 165 000/cm^3^. Metabolic panel revealed a creatinine 2.0 mg/dL, total bilirubin 1.6 mg/dL, aspartate aminotransferase 263 IU/L, alanine aminotransferase 893 IU/L, and alkaline phosphatase 192 IU/L. The international normalized ratio was 1.03. Creatinine kinase was 11 IU/L. Urinalysis was normal. Right upper quadrant ultrasound with Doppler revealed a normal biliary system with patent vasculature. Esophagogastroduodenoscopy revealed a trivial nonbleeding esophageal ulcer, and he was started on esomeprazole. Viral studies for Epstein-Barr Virus (EBV), Cytomegalovirus (CMV), Hepatitis B Virus (HBV), Hepatitis C Virus (HCV), Human Immunodeficiency Virus (HIV), and Herpes Simplex Virus (HSV) returned normal. Given evidence of reactive lymphocytes on the white blood cell differential, a peripheral smear was obtained. The atypical cells were felt by our hematologic pathologist to be most consistent with a viral process. He was discharged with a working diagnosis of hepatocellular liver injury due to viral-induced hepatitis or drug-induced hepatitis (secondary to supplements), with expected improvement over the next few weeks. He was discharged with close outpatient follow-up for his acute kidney injury.

**Figure 1.**
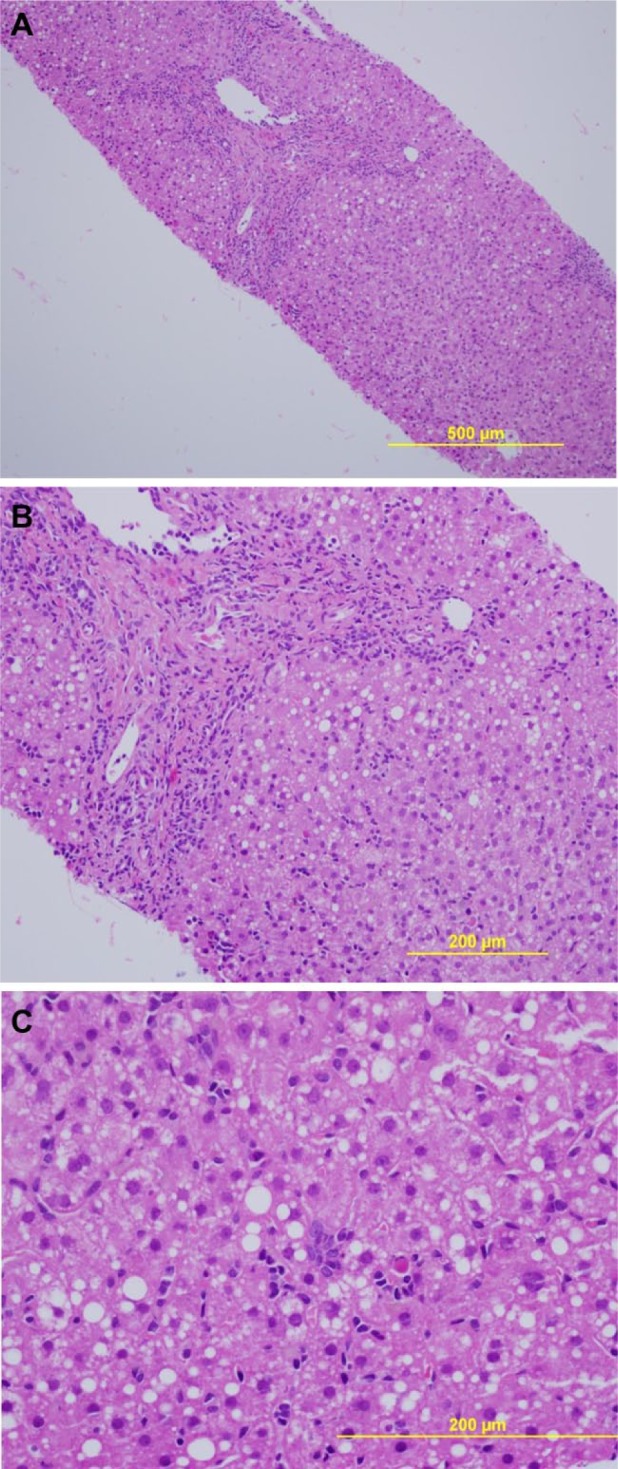
Liver biopsy. In (A) is shown liver stained with hematoxylin and eosin, revealing an inflammatory infiltrate primarily in the portal tract (upper left side of the image). In (B) is shown a portion of the same field at higher magnification, revealing the predominantly lymphocytic infiltrate, as well lymphocytes within the parenchyma. A small amount of fat can also be seen in hepatocytes. In (C) is shown the hepatic parenchyma, depicting lymphocytic infiltration, and several plasma cells as well as an acidophilic body.

**Figure 2.**
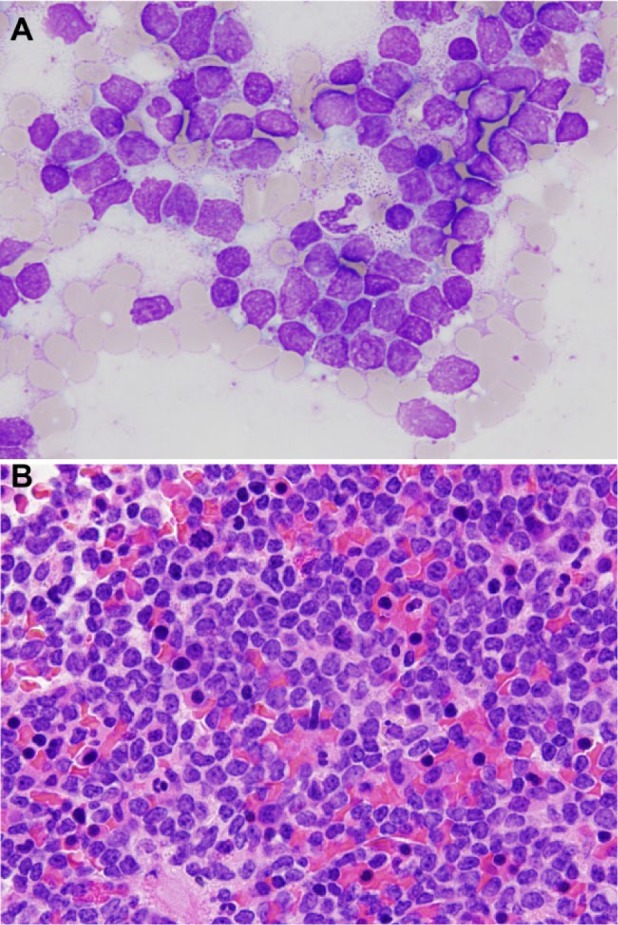
Bone marrow biopsy. In (A) is shown a bone marrow touch preparation demonstrating a predominance of intermediate sized cells with high nuclear to cytoplasmic ratios, fine nuclear chromatin, and variability prominent nucleoli, morphologically consistent with lymphoblasts. Wright-Giemsa, 50× magnification. In (B) is shown a bone marrow core biopsy revealing large aggregates of blasts with reduced numbers of red and white blood cell precursors, consistent with a decrease in erythropoiesis and myelopoiesis. Megakaryocytes were present in normal numbers. Hematoxylin and eosin, 50× magnification.

Six days after discharge, the patient re-presented with worsening of symptoms. He continued to describe nausea, vomiting, decreased PO intake, and progressive fatigue. Since discharge, he had lost another 10 pounds. Admission laboratory data revealed a white blood cell count of 17 320/cm^3^ (56% neutrophils, 17% lymphocytes, 10% reactive lymphocytes, 2% eosinophils), creatinine of 2.6 mg/dL, total bilirubin 2.6 mg/dL, aspartate aminotransferase 225 IU/L, alanine aminotransferase 869 IU/L, and alkaline phosphatase 191 IU/L. Given the persistent abnormalities in his liver tests, a liver biopsy was performed and revealed modest hepatocyte apoptosis and scattered portal and lobular inflammatory cells (primarily lymphocytes) felt to be consistent with drug-induced or viral hepatitis (Figure 1). Urine studies revealed a fractional excretion of sodium of 2.2%, suggesting intrinsic renal disease. Urinalysis with microscopy was benign with no evidence of cellular casts, dysmorphic red blood cells, white blood cells, or crystals. A kidney biopsy was performed to determine the cause of his acute kidney injury as the renal function did not improve with aggressive volume expansion. Two days after kidney biopsy, he developed pain in his left lower extremity, which on ultrasound was found to be caused by an extensive deep vein thrombosis encompassing the posterior tibial vein extending proximal up to the common femoral vein. Given his apparent hypercoagulable state, weight loss, and peripheral lymphocytosis, a hematologic malignancy was considered. Uric acid 12.3 mg/dL, lactate dehydrogenase 290 IU/L, phosphorus 5 mg/dL, and potassium 4.3 mmol/L. Peripheral blood flow cytometry revealed lymphoblasts, and bone marrow biopsy revealed 78% blasts with markers consistent with B-cell acute lymphoblastic leukemia (Figure 2). In retrospect, the atypical lymphocytes from the initial peripheral smear were actually lymphoblasts, although the blood smear was not available for re-review after the leukemia diagnosis. He was started on allopurinol and transferred to the inpatient malignant hematology service where he was started on induction chemotherapy.

## Discussion

Our patient was ultimately found to have acute B-cell lymphoblastic leukemia, a disorder of committed stem cells characterized by proliferation of immature lymphoblasts. Acute lymphoblastic leukemia (ALL) is the most common form of cancer in children, with the peak incidence occurring in young children aged 2 to 5 years. Survival rates in children approach 90%.^1-4^ In comparison, ALL is much less common in adults; ALL constitutes less than 20% of all acute leukemias in adults. Survival in adults is much poorer; estimated survival rates are between 20% and 40%.^2^ Common presenting signs and symptoms of ALL, although nonspecific, include fever and infection caused by neutropenia, bruising and bleeding secondary to thrombocytopenia, and fatigue and pallor due to anemia. Extramedullary tumor infiltration into the lymph nodes, spleen, testicles, and central nervous system are common.^1^

Based on the available literature and our clinical experience, our patient had an atypical presentation of ALL. The presentation was characterized by nausea, vomiting, and right upper quadrant abdominal pain with laboratory values consistent with hepatitis. Liver involvement has been reported to be a frequent finding in patients with ALL, and the key point is that it can present with a variety of syndromes ranging from asymptomatic hepatomegaly with infiltration of lymphocytes to hepatitis to acute liver failure.^3^ However, very different from our patient, the most common liver manifestation at the time of initial diagnosis is asymptomatic hepatomegaly.^4^

In contrast to the picture at presentation, hepatocellular injury is commonly observed in ALL patients during their treatment course secondary to liver injury from medications, post-chemotherapy viral infections (especially Hepatitis B), sepsis, and perhaps even ischemia.^4^ However, while hepatitis is a well-known complication during treatment of ALL, hepatocellular injury at the onset of diagnosis, as in our patient, is rare. Most of the literature describing liver disease in patients with ALL is in the pediatric population.^4^ In one retrospective pediatric study examining hepatitis at the time of ALL diagnosis, roughly one third of patients had increased aminotransferases with normal bilirubin and alkaline phosphatase (ie, laboratory values consistent with hepatitis) without evidence of viral hepatitis (negative HAV, HBV, HCV, HSV, EBV, and CMV). ALL-induced hepatitis was found to be more common in patients with T-cell ALL and in patients with higher white blood cell counts, uric acid levels, and lactate dehydrogenase levels, suggesting tumor infiltration as the underlying etiology. Further support stems from resolution of hepatitis in all subjects with treatment.^4^ The mechanism of hepatitis is unclear, but is likely to be either a paraneoplastic phenomenon (perhaps as in our patient) or due to lymphocyte infiltration of the liver (our patient had lymphocyte infiltration, but this was primarily in the lobule and was scattered, unlikely to be consistent with a large cell burden). Our patient’s abnormal liver function tests normalized after induction chemotherapy, consistent with either a paraneoplastic phenomenon or tumor infiltration as the underlying etiology. Of note, risk factors for hepatitis at the initial presentation of ALL include a high white blood cell count, older age, bulky disease, and T-cell leukemia.^4^

Our patient also had evidence of acute renal failure at the time of ALL diagnosis. According to published data, the incidence of renal failure in untreated ALL patients ranges from 13% to 25%.^5^ Etiologies for acute renal failure at the time of ALL diagnosis include leukemic infiltration of the kidney, spontaneous tumor lysis syndrome, and acute tubular necrosis. Leukemic infiltration is associated with significantly elevated white blood cell counts and T-cell leukemia and nephromegaly may be present on imaging.^5^ Spontaneous tumor lysis is another potential etiology for acute renal failure at initial presentation. It can occur prior to treatment with chemotherapy and presents as laboratory abnormalities including hyperuricemia, hyperkalemia, hyperphosphatemia, and hypocalcemia, along with one or a combination of clinical findings including acute kidney injury, arrhythmia, and seizures. Cell lysis results in a massive and rapid release of cellular components into the blood. In a retrospective study of 1072 adult inpatients with acute renal failure, 12 cases of spontaneous tumor lysis—4 with Burkitt lymphoma, 6 with non-Hodgkin lymphoma, 1 with retroperitoneal leiomyosarcoma, and 1 with B-cell ALL—were identified.^6^ Although it is most common with Burkitt lymphoma, spontaneous tumor lysis should be considered in patients with acute renal failure and ALL.^6^ Acute tubular necrosis is another potential etiology for acute renal failure at the time of diagnosis of ALL. Underlying etiologies include hypovolemia, sepsis, or nephrotoxic antibiotics. Our patient developed polyuria over the course of the following week, which led us to a diagnosis of post-ATN (acute tubular necrosis) diuresis. His urine output was matched with intravenous fluids and his renal failure slowly improved.

The appearance of an unprovoked deep vein thrombosis was a major clue for diagnosis of ALL in our patient. Unprovoked venous thromboembolism may be the earliest sign of cancer with up to 10% of patients with unprovoked venous thromboembolism receiving a new diagnosis of cancer in the following year.^7^ In patients with known hematologic malignancies, venous thromboembolism is a complication with an incidence of approximately 2%. The incidence between acute myeloid leukemia and ALL is comparable.^8^ Underlying etiologies for thrombosis in untreated malignancies include compression of veins by the tumor, immobilization, catheters, surgery, and thrombophilia.^9^ The hypercoagulable state of patients with leukemia is thought to be secondary to intravascular activation of the clotting system and upregulation of prothrombotic agents. Although the mechanism is unclear, there is evidence of increased thrombin generation in children with ALL at diagnosis, suggesting a potential etiology.^10^

In summary, although uncommon, ALL should be considered in the differential diagnosis of multisystemic organ involvement including acute liver and acute kidney injury, particularly in previously otherwise healthy individuals. Subtle clues to the diagnosis may include abnormal circulating cells and abnormal uric acid levels.
